# *Enterobacter asburiae* ST229: an emerging carbapenemases producer

**DOI:** 10.1038/s41598-024-55884-y

**Published:** 2024-03-14

**Authors:** Vittoria Mattioni Marchetti, Angela Kuka, Aurora Piazza, Stefano Gaiarsa, Cristina Merla, Mariangela Sottosanti, Patrizia Cambieri, Roberta Migliavacca, Fausto Baldanti

**Affiliations:** 1 Department of Clinical, Surgical, Diagnostic and Paediatric Sciences, University of Pavia, Pavia, Italy; 2 Microbiology and Virology Unit, IRCCS Fondazione Policlinico San Matteo, Pavia, Italy; 3 Specialization School of Microbiology and Virology, University of Pavia, Pavia, Italy; 4 IRCCS Fondazione Policlinico San Matteo, Pavia, Italy; 5 Unit of Anaesthesia and Intensive Care II, IRCCS Fondazione Policlinico San Matteo, Pavia, Italy

**Keywords:** *Enterobacter asburiae*, Epidemiology, Surveillance, Carbapenemase-producing, NDM-1, VIM-1, Low-risk pathogens, WGS, Preventive medicine, Bacterial genetics

## Abstract

*Enterobacter asburiae*, member of the *Enterobacter cloacae* complex (ECC) group, shows an increasing clinical relevance being responsible for infections like pneumonia, urinary tract infections and septicemia. The aim of the present study was the investigation of the genomic features of two XDR *E. asburiae* ST229 clinical strains co-carrying *bla*NDM-1 and *bla*VIM-1 determinants, collected in October 2021 and in June 2022, respectively. Two *E. asburiae* strains were collected from rectal swabs of as many patients admitted to the cardiopulmonary intensive care unit of Fondazione I.R.C.C.S. “Policlinico San Matteo” in Pavia, Italy. Based on the antibiotic susceptibility profile results, both isolates showed an XDR phenotype, retaining susceptibility only to fluoroquinolones. Both isolates shared identical resistome, virulome, plasmid content, and belonged to ST229, a rarely reported sequence type. They co-harbored *bla*NDM-1 and *bla*VIM-1 genes, that resulted located on transferable plasmids by conjugation and transformation. Moreover, both strains differed in 24 SNPs and showed genetic relatedness with *E. asburiae* ST709 and ST27. We described the first case of ST229 *E. asburiae* co-harboring *bla*NDM-1 and *bla*VIM-1 in Italy. This study points out the emergence of carbapenemases in low-risk pathogens, representing a novel challenge for public health, that should include such types of strains in dedicated surveillance programs. Antimicrobial susceptibility testing was carried out using Thermo Scientific™ Sensititre™ Gram Negative MIC Plates DKMGN. Both strains underwent whole-genome sequencing (WGS) using Illumina Miseq platform. Resistome, plasmidome, virulome, MLST, plasmid MLST and a SNPs-based phylogenetic tree were in silico determined.

## Introduction

*Enterobacter* species are Gram-negative, aerobic, and motile bacteria that belong to the *Enterobacteriaceae* family. *Enterobacter* spp. occurs ubiquitously in the environment, but it is also relevant from a clinical point of view, being responsible for infections like pneumonia, urinary tract infections and septicemia^1^. *Enterobacter* spp. is a member of the ESKAPE group, which is of particular concern, since infections caused by ESKAPE pathogens often results in the worsening of patient outcomes^2^. Despite the taxonomy of this genus is still a topic of debate, it is acknowledged that *Enterobacter cloacae* complex (ECC) represents a highly diverse bacterial group that includes, at present, twelve species and 22 clades^1,3^. The species grouped into the ECC are *Enterobacter asburiae, Enterobacter cloacae, Enterobacter hormaechei, Enterobacter kobei, Enterobacter ludwigii, Enterobacter pasteurii, Enterobacter xiangfangensis, E. bugandensis, E. cancerogenous, E. chengduensis, E. chuandaensis, E. sichuanensis, and E. roggenkampii* (Ref. ^4,5^ https://www.ncbi.nlm.nih.gov/Taxonomy/Browser/). Determining the correct ECC cluster is difficult but critical, since the proper classification holds clinical relevance. 16S rRNA gene sequencing was demonstrated to not provide sufficient discrimination for closely related species within the *Enterobacteriaceae* family, particularly within the ECC. The current methods showing a good discriminatory power in the proper identification of species within the ECC are the MALDI-TOF Biotyper, the online database coupled to an original Mass Spectrometric Identification (MSI) algorithm and Whole-Genome Sequencing (WGS) analysis^4,6^.

The rate of ECC infections has risen considerably, becoming a serious clinical issue over the last ten years. ECC species have become common nosocomial pathogens, with different ECC clusters showing differences in resistance mechanisms against β-lactams, colistin and some other antibiotics^1^.

Extended-spectrum β-lactamase (ESBL) genes have been increasingly detected in ECC isolates, particularly in nosocomial settings. Interestingly, ECC species shows a striking facility to acquire genetic elements like multi-class antibiotic resistance plasmids. The emergence of multidrug resistance (MDR), including resistance to the last-resort carbapenems, has led to an increased attention to in these organisms^7^. The transmission of antimicrobial resistance (AMR) traits is attributable to the horizontal gene transfer (HGT) by conjugative plasmids that are able to acquire several AMR islands and spread AMR across species^8^. As a result, MDR and extensively drug-resistant (XDR) bacteria rapidly emerged and have limited the available therapeutic options against common infections, increasing rates of morbidity and mortality^9^.

The objective of this study was to investigate the genomic features of two XDR *E. asburiae* clinical strains co-carrying *bla*NDM-1 and *bla*VIM-1 determinants.

## Methods

### Bacterial isolates and antimicrobial susceptibility testing

Clinical samples were plated on CHROMID^®^ Carba (Biomerieux, Marcy l'Etoile, France), a selective chromogenic medium for all carbapenemase-producing *Enterobacteriaceae*. Strains were identified at the genus and species level by Matrix-Assisted Laser Desorption Ionization Time-Of-Flight Mass Spectrometry (MALDI-TOF MS) (Bruker Daltonics GmbH, Bremen, Germany) and analyzed using BioTyper version 3.0. The NG-Test^®^ CARBA-5 (NG Biotech laboratories) immunochromatographic assay was used to confirm carbapenemase production and identify the enzyme class responsible for the resistant phenotype. Antimicrobial susceptibility testing (AST) was carried out according to standard internal laboratory protocol using Thermo Scientific™ Sensititre™ Gram Negative MIC Plates DKMGN, an antibiotic susceptibility test by broth microdilution. MIC values were interpreted according to the European Committee on Antimicrobial Susceptibility Testing (EUCAST) breakpoints (The European Committee on Antimicrobial Susceptibility Testing. Breakpoint tables for interpretation of MICs and zone diameters http://www.eucast.org).

### Whole-genome sequencing (WGS)

Both strains underwent Whole-Genome Sequencing (WGS). The genomic DNA, extracted using the DNeasy Blood&Tissue kit (Qiagen), was sequenced on an Illumina Miseq platform (Illumina Inc., San Diego, CA, USA) with a 250-bp paired-end sequencing, after library preparation with the Nextera XT library preparation kit. Reads were quality-checked with FastQC and then assembled within the Shovill^10^ pipeline. Assembled sequences were annotated using Prokka and manually checked by Rapid Annotation using Subsystems Technology (RAST server)^11,12^. The mean nucleotide identity of the two studied strains was conducted using FastANI v1.34 (https://github.com/ParBLiSS/FastANI) against the reference genome for *E. asburiae* (GenBank accession number: GCA_007035805.1), *E. cloacae* (GenBank accession number: GCA_905331265.2), *E. pasteurii* (GenBank accession number: GCA_014930725.1) and *E. xiangfangensis* (GenBank accession number: GCA_001729785.1).

Resistome, plasmid replicon content, MLST and plasmid MLST (pMLST) were determined through uploading the assembled sequences to ResFinder 4.1 and PlasmidFinder, MLST and pMLST, respectively, available at the Center for Genomic Epidemiology website (https://www.genomicepidemiology.org/). A sequence-based typing analysis on the *E. asburiae* MLST allelic profiles (ECC MLST scheme; https://pubmlst.org/organisms/enterobacter-cloacae) was generated using the PHYLOViZ Online Tool (https://online.phyloviz.net/index).

The virulence content was obtained using both RAST and Virulence Factor Database (VFDB)^13^. Comparative genome alignment was conducted with SnapGene software (https://www.snapgene.com/) and Mauve^14^.

### SNP analysis and phylogenetic reconstruction

Phylogenetic relationship between the two studied strains and global genomes were investigated. A total of 479 *E. asburiae* genomes were retrieved from the NCBI database, including both complete and draft genomes. The map of the global epidemiology of the 481 *E. asburiae* genomes and timeline representation were drawn using the online mapchart tool (https://www.mapchart.net/) and the online visualizer Microreact (https://microreact.org/), respectively. Phylogenetic trees were obtained using single nucleotide polymorphisms (SNPs) obtained with parsnp v1.2 (https://github.com/marbl/parsnp/) and genome ASM3058080v1 as reference. Graphic illustrations of the tree were built with the interactive tree of life (iTOL) (https://itol.embl.de/). Based on the global phylogeny through parsnp v1.2, thirteen genomes, including the two studied *E. asburiae*, were selected and compared for resistome, plasmidome and virulome. The evaluation of the number of SNPs was conducted on the thirteen selected genomes through CSI phylogeny 1.4 available at https://cge.food.dtu.dk/services/CSIPhylogeny/^15^.

### Pangenome analysis

Pangenome evaluation was conducted with the Roary pipeline^16^, with default parameters. The gene presence/absence file from the Roary analysis was used to visualize the distributions for individual isolates, obtained via Python3^17^.

### Conjugation/transformation assay

The transferability of *bla*NDM-1 and *bla*VIM-1 genes was tested in liquid medium using *E. coli* J62 strain (SM^R^) as recipient. Transconjugants were selected on MacConkey agar plates (Scharlab, SL, Barcelona, Spain) containing streptomycin (1000 mg/L) (Sigma-Aldrich, St. Louis, MO, United States) and cefotaxime (16 mg/L) (Sigma-Aldrich). Furthermore, transformation was carried out with both *E. asburiae*; plasmid extraction was performed using ZymoPURE Plasmid Miniprep Kit (Zymo research), and chemically competent Top10 cells were used as the recipient. Transformants were selected on Mueller-Hilton (MH) agar (Oxoid, Hampshire, UK) with 100 mg/L Ampicillin (Sigma-Aldrich). The presence of *bla*NDM-1 and *bla*VIM-1 in the transconjugants/transformants and the typing of the plasmids were confirmed by PCR and PCR replicon typing (PBRT 2.0 kit), respectively.

### Epidemiological map

Epidemiological representation of the 481 *E. asburiae* genomes, including the two *E. asburiae* here studied, was constructed using Microreact tool, pointing out data on MLST, year and region of isolation^18^.

### Data availability

The nucleotide sequences of the two genomes were deposited and are available in GenBank under the Bioproject ID PRJNA1043153.

### Ethical-statement

The study was designed and conducted in accordance with the Helsinki Declaration and approved by the Ethics Committee of Fondazione IRCCS Policlinico San Matteo in Pavia, Italy (Fasc. 2023-3.11/105). The work described herein is a retrospective study performed on bacterial isolates from human samples that were obtained as part of routine hospital care. An informed consent was signed by all the patients as part of the hospital routine activity, and accordingly to “Comitato Etico Territoriale Lombardia 6”.

## Results

### Clinical data

Two strains of *E. asburiae* were collected from the Cardiopulmonary Intensive Care Unit (ICU) of Fondazione I.R.C.C.S. “Policlinico San Matteo” in Pavia, Italy, a 900-bed hospital. The two isolates were obtained in October 2021 and in June 2022 from rectal swabs of two different patients.

Patient 1 was a 59-year-old male, admitted to the Cardiopulmonary ICU in September 2021, after cardiac surgery (aortic acute dissection). Surveillance rectal swab collected at admission tested negative for the presence of carbapenemase-producing *Enterobacteriaceae*. One week after admission (day 7), the patient developed pneumonia, caused by multi-susceptible strains of *Klebsiella pneumoniae* and *Escherichia coli*. The patient was treated with vancomycin and piperacillin/tazobactam. On day 17, bronchoaspirate examination resulted negative for the presence of pathogenic bacteria. However, on day 25 bronchoaspirate test revealed the presence of carbapenem-resistant *Pseudomonas aeruginosa*. After this isolation, antibiotic therapy was changed to linezolid and meropenem until antimicrobial resistance characterization and then converted to amikacin and ceftazidime-avibactam once carbapenem resistance was determined. On day 35, a surveillance rectal swab was tested positive for the presence of NDM- and VIM-producing *E. asburiae* (6370). On day 36, therapy was changed to cefiderocol. On day 37 of Cardiopulmonary ICU hospitalization, a rectal swab was tested positive for the presence of NDM-producing *K. pneumoniae*, NDM-producing *E. coli*, and *E. asburiae* producing both VIM and NDM. Colonization with all three strains persisted until the patient's death (day 46) but did not evolve into infection. Other specimens, such as urine, bronchoaspirate, blood cultures, and sternal wound swabs, were negative for the presence of *E. asburiae*. On day 46, the patient died due to multi-organ failure.

Patient 2 was a 59-year-old male, admitted to the Cardiopulmonary ICU in May 2022. Patient was assisted in the emergency department following a syncope event and underwent surgery for myocardial revascularization. During the first two months of hospitalization, the patient developed pneumonia caused by *Candida tropicalis* and ESBL-producing *Proteus mirabilis* and a urinary tract infection by multi-susceptible *Morganella morganii* and *P. aeruginosa*. Upon admission, surveillance rectal swab for carbapenemase-producing *Enterobacteriaceae* resulted negative. On day 42, rectal swab screening revealed the presence of NDM- and VIM-producing *E. asburiae* (7108). Multiple antibiotics were administered to the patient during his Cardiopulmonary ICU hospitalization: vancomycin (day 1–8; day 29–39), piperacillin/tazobactam (day 1–9), meropenem (day 9–16; day 28–36; day 52 to discharge), caspofungin (day 8–18), ceftaroline (Day 10–19), and amikacin (day 52 to discharge). On day 58 of Cardiopulmonary ICU hospitalization, the patient was transferred in stable condition to the intensive care unit of another hospital in the province of Pavia (Voghera) with ongoing antibiotic therapy (meropenem and amikacin).

### Identification, antimicrobial resistance and WGS analyses on resistome, virulome and plasmidome

The BioTyper MALDI-TOF MS identification assigned 6370 and 7108 isolates to *E. asburiae* species with a 99.99% confidence. This result was confirmed by FastANI analysis showing a 98.5% identity with *E. asburiae* species for both the studied strains.

Based on the antibiogram results, both isolates showed an XDR phenotype, retaining susceptibility only to fluoroquinolones (Table 1). The resistome analysis through WGS on both isolates unveiled the presence of resistance genes for aminoglycosides (*aac(6′)-Ib-cr*, *aadA1*, *aph(3′)-XV*, *rmtC*), macrolides (*mph(A)*), trimethoprim (*dfrA14*), sulphonamides (*sul1*), chloramphenicols (*catB2*), antiseptics (*qacE*), and β-lactams (*bla*SHV-12, *bla*NDM-1, *bla*VIM-1).

**Table 1:** Resistance profile, resistome, Inc groups, pMLST and MLST of the two *E. asburiae.*

	*E. asburiae* 6370	*E. asburiae* 7108
Resistance profile	AMP, AMS, ATM, CTX, TET, CAZ, COL, TZP, ETP, MEM, IMI, GEN, TOB, TGC, SXT, PIP	AMP, AMS, ATM, CTX, TET, CAZ, COL, TZP, ETP, MEM, GEN, TOB, TGC, SXT, PIP
Resistome	*rmtC, aph(3′)-XV, aadA1, aac(6′)-Ib3, bla*ACT-10*, bla*NDM-1*, bla*VIM-1*, bla*SHV-12*, mph(A), catB2, fosA, sul1, dfrA14*	*rmtC*, *aph(3′)-XV*, *aadA1*, *aac(6′)-Ib3*, *bla*ACT-10, *bla*NDM-1, *bla*VIM-1, *bla*SHV-12, *mph(A)*, *catB2*, *fosA*, *sul1*, *dfrA14*
Inc groups	IncA/C, IncFIB, IncFII (Yp), IncFII (pECLA), IncN, IncR	IncA/C, IncFIB, IncFII (Yp), IncFII (pECLA), IncN, IncR
pMLST	IncA/C ST12, IncF Y4:A-:B36, IncN-	IncA/C ST12, IncF Y4:A-:B36, IncN-
MLST	ST229	ST229

AMP, ampicillin; AMS, ampicillin/sulbactam; ATM, aztreonam; CTX, cefotaxime; TET, tetracycline; CAZ, ceftazidime; COL, colistin; TZP, piperacillin-tazobactam; ETP, ertapenem; MEM, meropenem; IMI, imipenem; GEN, gentamycin; TOB, tobramycin; TGC, tigecycline; SXT, trimethoprim/sulfamethoxazole; PIP, piperacillin.

**Figure 1:**
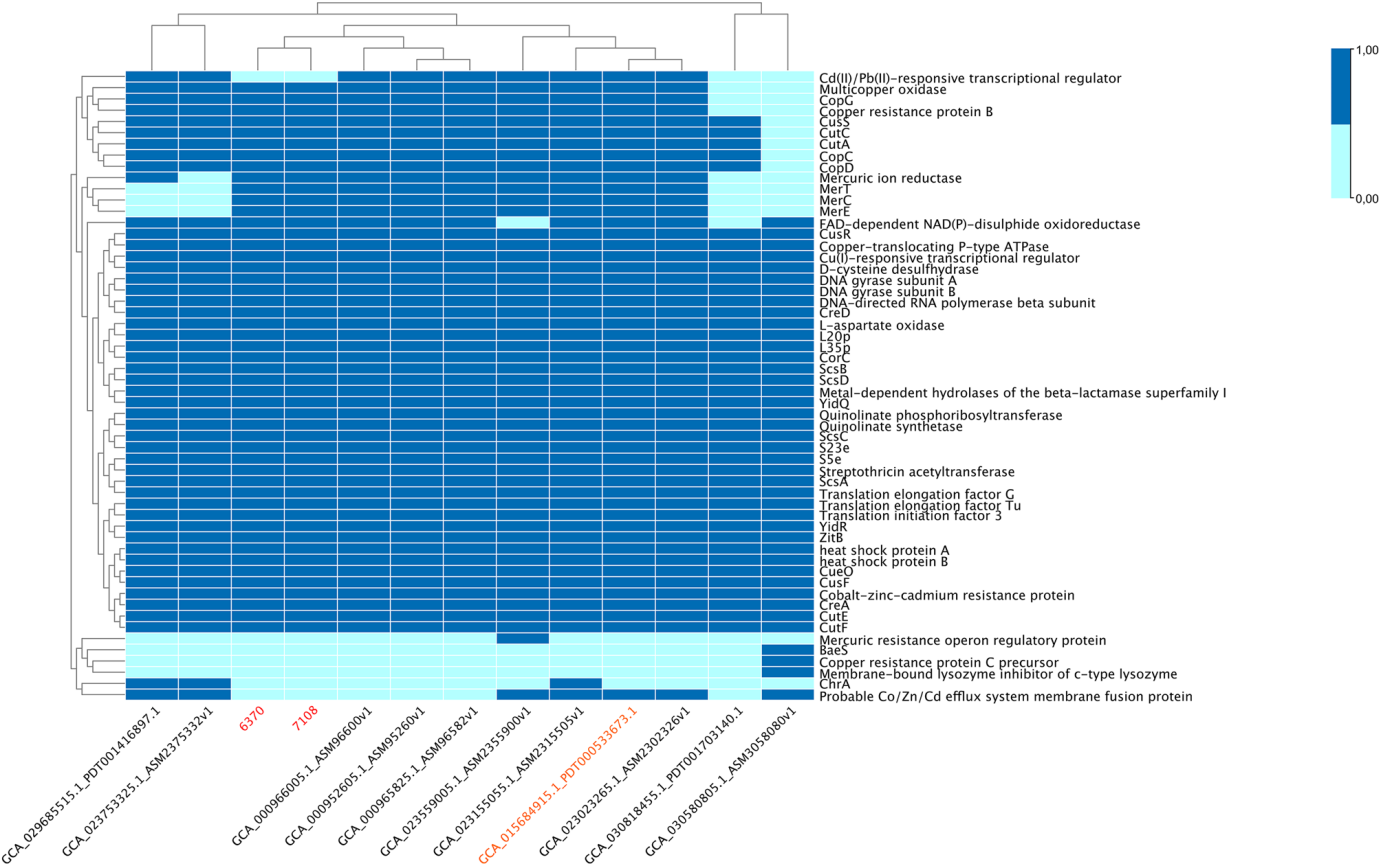
Heatmap of the virulence genes content of the thirteen *E. asburiae* strains, including the two objects of the study (in red), clonally related. In orange is reported the further ST229 *E. asburiae* strain (accession number PDT000533673.19).

Based on the virulome content detected by RAST and VFDB, the three *E. asburiae* ST229 strains in analysis, 6370 and 7108 here described, and PDT000533673.19 from GenBank, harbored genes involved in adhesion, invasion, tolerance to colicin E2, copper homeostasis/tolerance, cobalt-zinc-cadmium resistance and a mercury resistance operon (Fig. 1). The virulome of the three genomes was identical, except for PDT000533673.19, which additionally carried Cd(II)/Pb(II), and a Co/Zn/Cd efflux system. The virulome comparison among the thirteen studied genomes, revealed a shared virulence genes content, in particular of the heat shock proteins and several components involved in copper and mercury homeostasis (Fig. 1).

The plasmidome investigation highlighted the prevalence of IncA, IncFIB, IncFII, IncN and IncR plasmids in both isolates (Table 1).

### Genetic context of the blaNDM-1 and blaVIM-1

Based on in silico analysis, the *bla*NDM-1 gene was located in a composite transposon of 10 Kb flanked by ΔIS*Aba125* and IS*26*. The BLAST-based analysis (nt database) of the genomic island revealed identical match (query and identity 100%) to the reference of the largely described NDM cassette (Accession number: KF220657), involved in the successful spread of different NDM variants^19^. Similarly, *bla*VIM-1 determinant was flanked by *IntI*1 and followed by *aac(6′)-Ib-cr, aph(3′)-II, aadA, catB, qacE* and *sul1*. This cassette, of 6 Kb, shared identity (query and identity 100%) with international VIM-carrying plasmids, isolated from different *Enterobacterales* (Accessions numbers: CP050069, CP034084.1, CP132210.1, OQ111274).

### Conjugation and transformation assays

According to both conjugation and transformation results, the *bla*NDM-1 gene was successfully transferable by both the *E. asburiae* strains. Conversely, the *bla*VIM-1 resulted transferable only for the 6370 isolate (Table 2).

**Table 2:** Antibiogram, antimicrobial resistance genes and replicons results of conjugation and transformation.

ID	Species	AK	AMC	AMP	ATM	FEP	CTX	CAZ	CIP	COL	ERT	CN	LEV	MEM	TZP	TOB	AMR	Replicon
J62	*E. coli*	< = 8 S	< 32 S	< 8 S	< 1 S	< 8 S	< 32 S	< 32 S	< 0.06 S	< 2 S	0.25 S	< 2 S	< 0.5 S	< 2 S	< 8 S	< 2 S	ND	ND
TOP10	*E. coli*	< = 8 S	< 32 S	< 8 S	< 1 S	< 8 S	< 32 S	< 32 S	< 0.06 S	< 2 S	0.25 S	< 2 S	< 0.5 S	< 2 S	< 8 S	< 2 S	ND	ND
6370	*E. asburiae*	> 16 R	> 32 R	> 8 R	> 16 R	> 8 R	> 32 R	> 32 R	< 0.06 S	> 4 R	> 1 R	> 4 R	< 0.5 S	> 32 R	> 16 R	> 4 R	*bla*NDM, *bla*VIM	IncA/C, IncN
J62*6370	*E. coli*	< = 8 S	> 32 R	> 8 R	4 I	> 8 R	> 32 R	> 32 R	< 0.06 S	< 2 S	> 1 R	> 4 R	< 0.5 S	> 32 R	> 16 R	> 4 R	*bla*NDM, *bla*VIM	IncA/C, IncN
TOP10*6370	*E. coli*	< = 8 S	> 32 R	> 8 R	4 I	> 8 R	> 32 R	> 32 R	< 0.06 S	< 2 S	> 1 R	> 4 R	< 0.5 S	> 32 R	> 16 R	> 4 R	*bla*NDM, *bla*VIM	IncA/C, IncN
7108	*E. asburiae*	> 16 R	> 32 R	> 8 R	> 16 R	> 8 R	> 32 R	> 32 R	< 0.06 S	> 4 R	> 1 R	> 4 R	< 0.5 S	> 32 R	> 16 R	> 4 R	*bla*NDM, *bla*VIM	IncA/C, IncN
J62*7108	*E. coli*	> 16 R	> 32 R	> 8 R	< = 1 S	> 8 R	> 32 R	> 32 R	< 0.06 S	< 2 S	> 1 R	> 4 R	< 0.5 S	16 R	> 16 R	> 4 R	*bla*NDM	IncN
TOP10*7108	*E. coli*	> 16 R	> 32 R	> 8 R	< = 1 S	> 8 R	> 32 R	> 32 R	< 0.06 S	< 2 S	> 1 R	> 4 R	< 0.5 S	> 32 R	> 16 R	> 4 R	*bla*NDM	IncN

AK: amikacin; AMC: amoxicillin/clavulanic acid; ATM: aztreonam; FEP: cefepime; CTX: cefotazime; CAZ: ceftazidime; CIP: ciprofloxacin; COL: colistin; ERT: ertapenem; CN: gentamicin; LEV: levofloxacin; MEM: meropenem; TZP: piperacillin/tazobactam; TOB: tobramycin.

AMR: antimicrobial resistance genes; ND: not detected.

### Genetic relatedness

**Figure 2:**
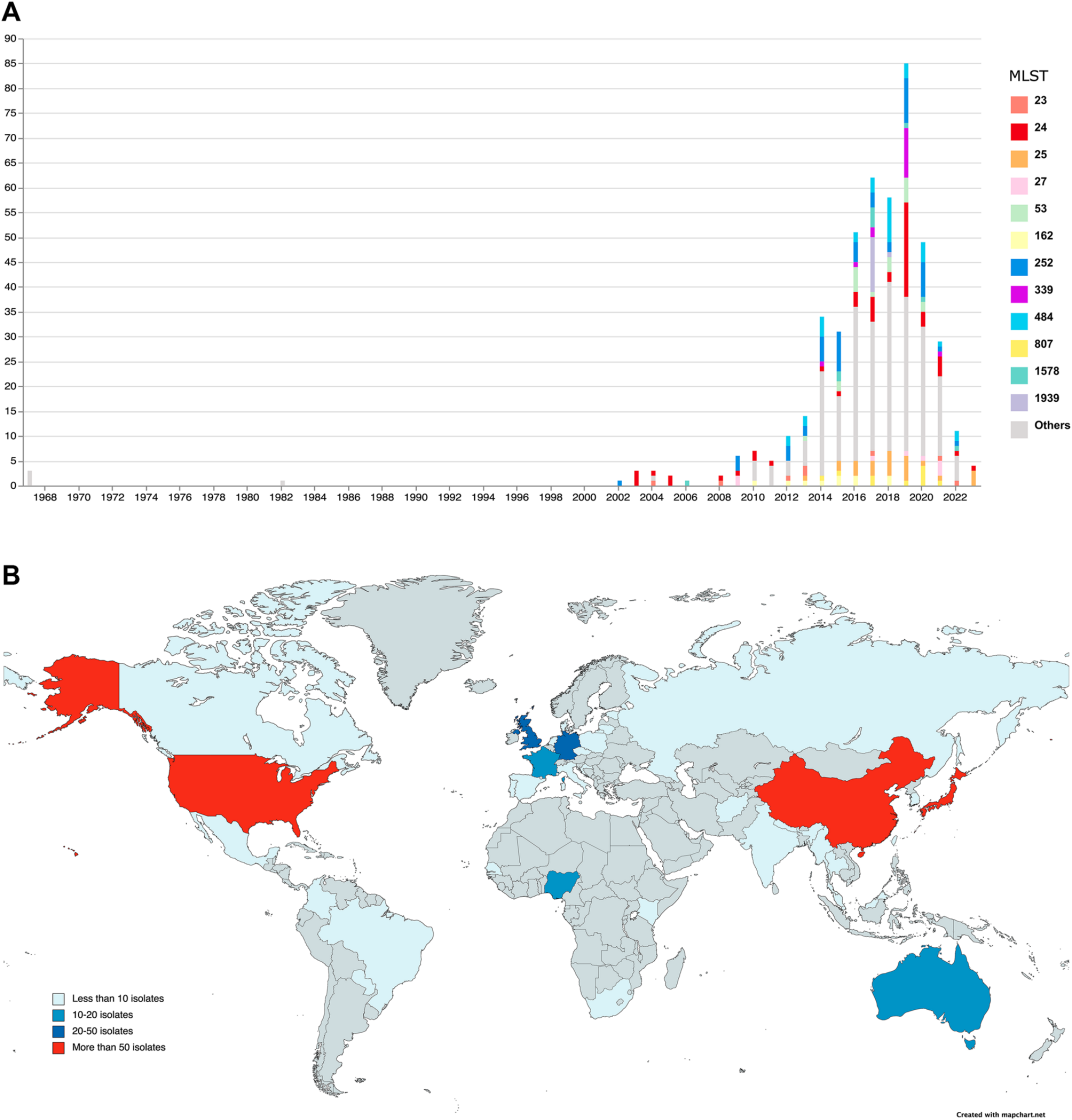
(**A**) timeline of the date of collection of the 481 *E. asburiae* (https://microreact.org/). (**B**) epidemiological map of the MLST of the 481 *E. asburiae* genomes obtained from the NCBI database (https://www.mapchart.net/). The dimension of the circle is related with the number of genomes associated to a particular region of origin.

**Figure 3:**
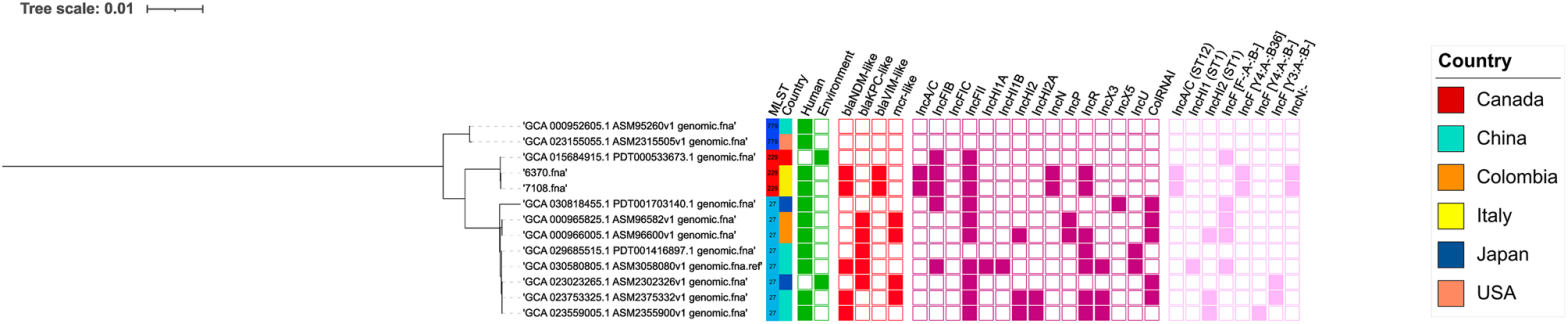
iTOL v6 raffiguration of SNPs-based tree for the 13 selected genomes obtained by parsnp and the related genome content. Green grid = source of isolates; red grid = carbapenem- and colistin-resistance genes; purple grid = plasmid content; pink grid = pMLST.

A sequence-based typing analysis on the *E. asburiae* MLST allelic profiles (ECC MLST scheme; https://pubmlst.org/organisms/enterobacter-cloacae) assigned ST229 to both isolates. Based on the PubMLST alleles scheme only, a PHYLOViZ investigation highlighted a correlation of ST229 with the sequence type ST27 and ST709 (Supplementary Fig. 1). The NCBI database (accessed in August 2023) contains 479 genomes classified as *E. asburiae*. Since 2008, *E. asburiae* has been reported worldwide, with an increased number of isolates recorded in 2019. Based on MLST scheme, the geographical distribution of lineages is heterogeneous, with a predominance of ST24 in Asia, Europe, Central/South America and Nigeria, and ST252 in Asia, Europe and Central America (Fig. 2A, B). To deepen the genomic relatedness among global isolates, a SNPs based phylogeny analysis was conducted (Supplementary Fig. 2). The two *E. asburiae* strains were compared against the 479 genomes found in the NCBI database. Interestingly, the SNP-based phylogeny revealed high identity between the two ST229 *E. asburiae*, which clustered together with another ST229 *E. asburiae* (PDT000533673.19) collected in 1982 from the Canadian wildlife environment (Fig. 2A, B). The Canadian genome possessed a narrow resistome consisting of only *bla*ACT-10 and *fosA* genes. The ST229 cluster showed genetic relatedness with a ST709 (ASM95260v1, ASM2315505) and a ST27 cluster (PDT001703140.1, ASM96582v1, ASM9660v1, PDT001416897, ASM3058080v1, ASM2302326v1, ASM2375332v1, ASM2355900v1), as suggested by the PHYLOVIZ analysis (Fig. 3). All the 13 genomes, belonging to the three different STs, were analyzed by pan-genome-based (Roary) and SNP-based (CSI) phylogeny, which confirmed the aforementioned relatedness. In particular, pan-genome representation pointed out an overall number of 9237 genes: a core genome composed of 3529 genes, and a soft-core of 5708 (3090 genes belonging to the shell and the remaining 2618 genes to cloud) (Supplementary Fig. 3A–B). In accordance with variant calling results, *E. asburiae* 6370 and *E. asburiae* 7108 differed for 24 SNPs, while the Canadian genome carried 1048 and 1060 SNPs, when compared with *E. asburiae* 6370 and 7108, respectively (Supplementary Table S1).

## Discussion

ECC is the third most relevant drug-resistant pathogen among *Enterobacterales* due to its ability to acquire successful resistance genes via horizontal gene transfer^20^. *E. asburiae* is a secondary member of the ECC and it is difficult to differentiate from *E. cloacae* by MALDI-TOF–MS only ^21^. Recent studies demonstrated an improved capability in *E. asburiae* identification by using MALDI-TOF–MS Biotyper coupled with online database and an original Mass Spectrometric Identification (MSI) supervised algorithm^4,6^; nonetheless they are not routinely used in clinical practice. Based on the current literature, *E. asburiae* is rarely reported in clinical infection events but, in accordance with the metadata of all the *E. asburiae* genomes available in NCBI, it is plausible to confirm of its spread and stabilization even in human samples^22^.

Since its first report in 1986, *E. asburiae* has been poorly associated with resistance to carbapenems^23^. The first case of carbapenems-resistance among *E. asburiae* emerged in 2005 from an environmental sample (river) in the USA, and then a similar phenotype was thoroughly described in 2017, when the IMI-2-carrying plasmid was detected in a clinical *E. asburiae* isolate from the Czech Republic^24,25^. Recently, another *bla*IMI-2-carrying *E. asburiae* was isolated in Sweden from a feed mill sample^26^, while the co-occurrence of carbapenem and colistin resistance has been described in a South Korean hospital^27^. The metallo-β-lactamases NDM-like enzymes efficiently hydrolyze a broad range of β-lactam antibiotics, and are well-disseminated worldwide, as in India, China, Nepal, or Near East^19^. VIM-like enzymes are other members of the metallo-β-lactamase family that have been frequently documented in ECC^28^. Cases of NDM and VIM enzymes among *E. asburiae* species are poorly reported in literature.

In the NCBI database are deposited only six genomes of *E. asburiae* (Accession numbers: GCA_030580805.1, GCA_026178845.2, GCA_022543975.1, GCA_022544075.1, GCA_022544455.1, GCA_022544255.1) harboring more than one carbapenemase but the co-expression of NDM- and VIM-like carbapenemases has not been reported in clinical *E. asburiae* strains yet. Here, we underline the first report of co-presence of *bla*NDM-1 and *bla*VIM-1 carbapenemases genes in two different patients colonized by *E. asburiae* ST229.

ST229 is an understudied ST that has not been described in the literature, so far. Noteworthy, the only *E. asburiae* ST229 available in NCBI database, was obtained in Canada from a 1982 environmental, and carries a very limited set of antimicrobial resistance genes. ST229 shared genomic relatedness with ST27 and ST709, not yet described in literature. ST27 and ST229 shared not only the ability to harbor widely spread resistance genes, but even to fit different groups of plasmids, with the IncFII as the predominant plasmid type. Moreover, the three STs shared several virulence genes, involved in copper homeostasis and adhesion, strengthening the hypothesis of a close genetic relation.

In accordance with our results, the detection of several well-known Inc group plasmids highlights the capability of ST229 to efficiently acquire the most notorious plasmids involved in the spread of relevant antimicrobial resistance genes. Indeed, IncA/C ST12 has been already associated to VIM-1 spread in Italy^29^, while IncHI1/2 ST1 are related with the CTX-M-type dissemination^30^. Moreover, the subtype IncF Y4:A-:B36 is related with *bla*NDM-1, *sul1* and *rmtC*^31^. Copper is a micronutrient indispensable for certain biological processes but needs to be mitigated by bacteria in order to avoid its toxic activity^32^. As demonstrated for *E. coli* and *Salmonella enterica*, copper has a relevant role in survival and invasion of the host; thus we speculate the possibility of a similar advantage for *E. asburiae*. No data are available on the implication of copper systems in the overall virulence of *E. asburiae*.

ST229 showed not only the ability of acquiring and fitting relevant plasmid-mediated-carbapenemase-genes, but even the capability to hold several virulence genes that can contribute to the adhesion and bacterial survival in unfavorable conditions. Altogether, our findings indicate ST229 *E. asburiae* as a possible novel *reservoir* of carbapenemases, as already speculated in other low risk pathogens^33^. The current knowledge on MDR and XDR *E. asburiae*, and particularly carbapenemase-producing *E. asburiae*, is incomplete and needs further investigations.

In conclusion, we described the first case of ST229 *E. asburiae* co-harboring *bla*NDM-1 + *bla*VIM-1 determinants from an Italian hospital. This study points out the importance of a correct species identification among members of ECC, in order to clarify the epidemiology and the impact of *E. asburiae*, especially carbapenemase-producing strains *E. asburiae*, in clinical settings. The emergence of carbapenemases in low risk pathogens could represent a novel challenge for public health, that should include such strains in dedicated surveillance programs.

### Supplementary Information

Supplementary Figure 1.

Supplementary Figure 2.

Supplementary Figure 3.

Supplementary Table S1.

Supplementary Legends.
