# Publisher Correction: *Enterobacter asburiae* ST229: an emerging carbapenemases producer

**DOI:** 10.1038/s41598-024-60104-8

**Published:** 2024-05-01

**Authors:** Vittoria Mattioni Marchetti, Angela Kuka, Aurora Piazza, Stefano Gaiarsa, Cristina Merla, Mariangela Sottosanti, Patrizia Cambieri, Roberta Migliavacca, Fausto Baldanti

**Affiliations:** 1 Department of Clinical, Surgical, Diagnostic and Paediatric Sciences, University of Pavia, Pavia, Italy; 2 Microbiology and Virology Unit, IRCCS Fondazione Policlinico San Matteo, Pavia, Italy; 3 Specialization School of Microbiology and Virology, University of Pavia, Pavia, Italy; 4 IRCCS Fondazione Policlinico San Matteo, Pavia, Italy; 5 Unit of Anaesthesia and Intensive Care II, IRCCS Fondazione Policlinico San Matteo, Pavia, Italy

Correction to: *Scientific Reports* 10.1038/s41598-024-55884-y, published online 14 March 2024

The original version of this article contained errors in the spelling of the authors Vittoria Mattioni Marchetti, Angela Kuka, Aurora Piazza, Stefano Gaiarsa, Cristina Merla, Mariangela Sottosanti, Patrizia Cambieri, Roberta Migliavacca & Fausto Baldanti, which were incorrectly given as Mattioni Marchetti Vittoria, Kuka Angela, Piazza Aurora, Gaiarsa Stefano, Merla Cristina, Sottasanti Mariangela, Cambieri Patrizia, Migliavacca Roberta & Baldanti Fausto respectively.

The original article has been corrected.

