# Supplementary figures and images for: Enterobacter asburiae ST229: an emerging carbapenemases producer
Source: Sci Rep. 2024 Mar 14;14:6220. doi: 10.1038/s41598-024-55884-y (PMC10940580; doi:10.1038/s41598-024-55884-y)

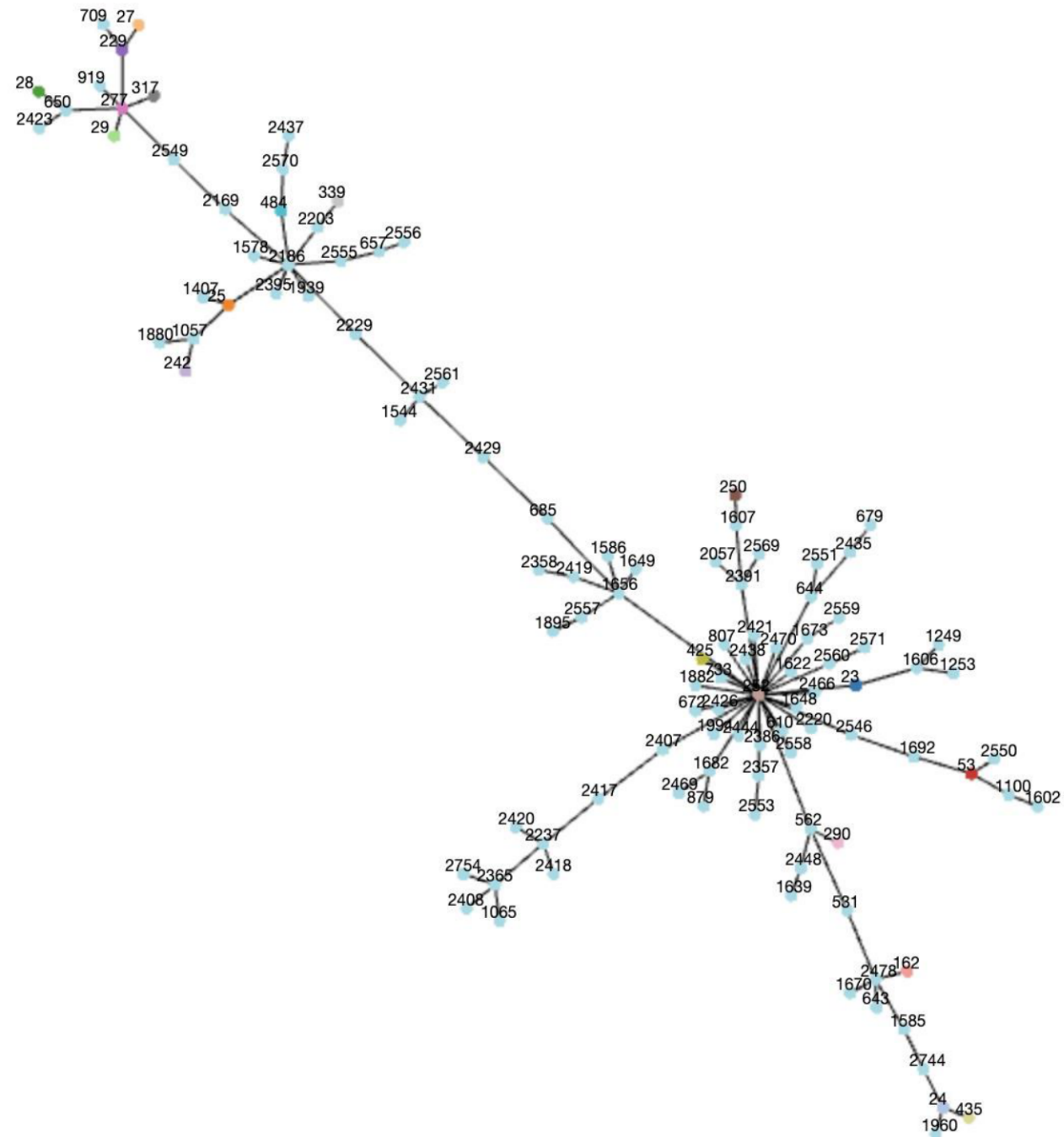

Supplement: Supplementary file 1 — Supplementary Figure 1. [file 41598_2024_55884_MOESM1_ESM.pdf]

Tree scale: 0.1

Source

- Human
- Animal
- Water
- Environment
- Food
- NA

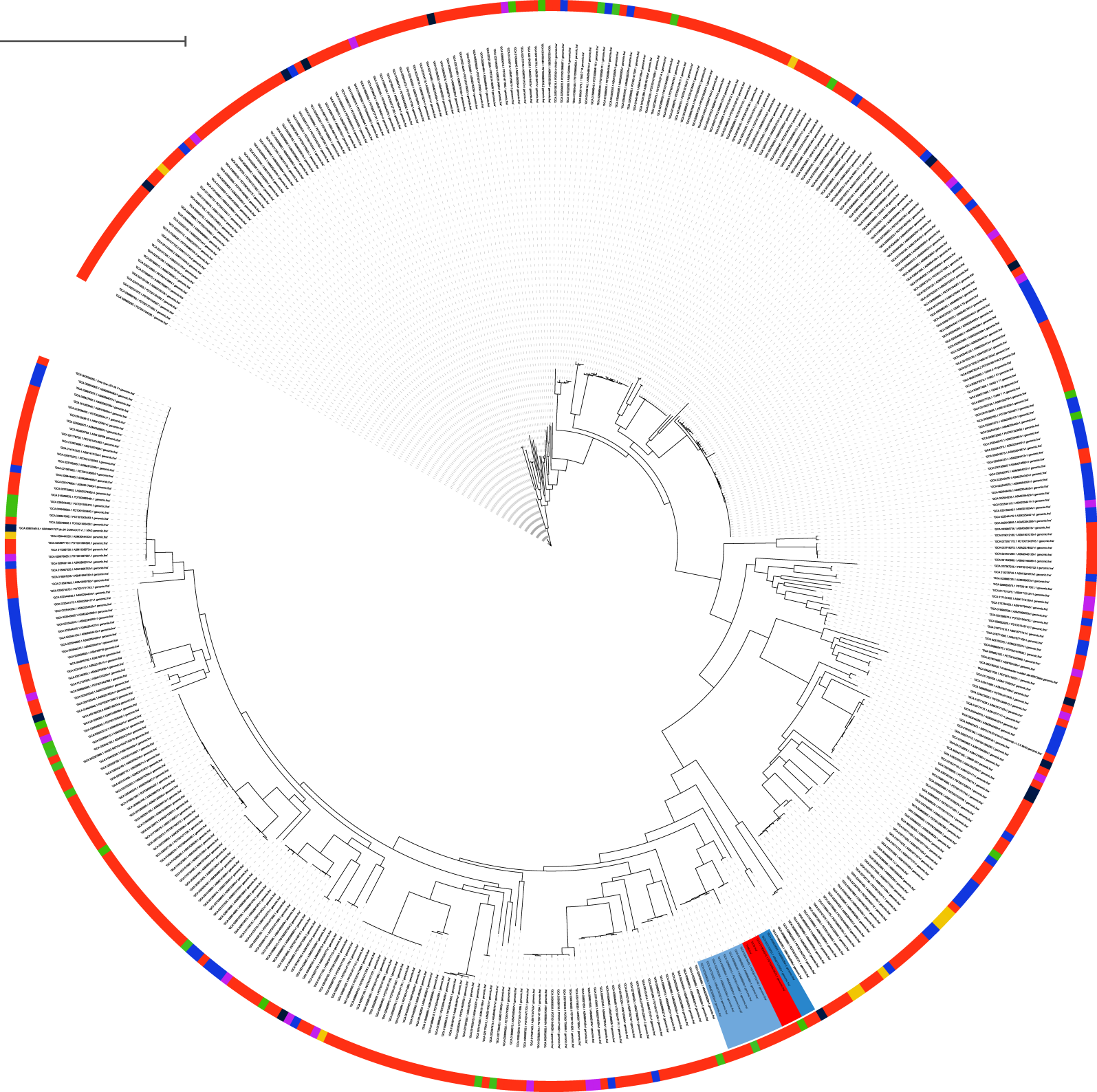

Supplement: Supplementary file 2 — Supplementary Figure 2. [file 41598_2024_55884_MOESM2_ESM.pdf]

Roary matrix  
(9237 gene clusters)

ASM3058080  
PDT0014168  
ASM96600v1  
PDT0017031  
PDT0005336  
6370  
7180  
ASM96582v1  
ASM2315505  
ASM95260v1  
ASM230236v  
ASM2355900  
ASM2375332

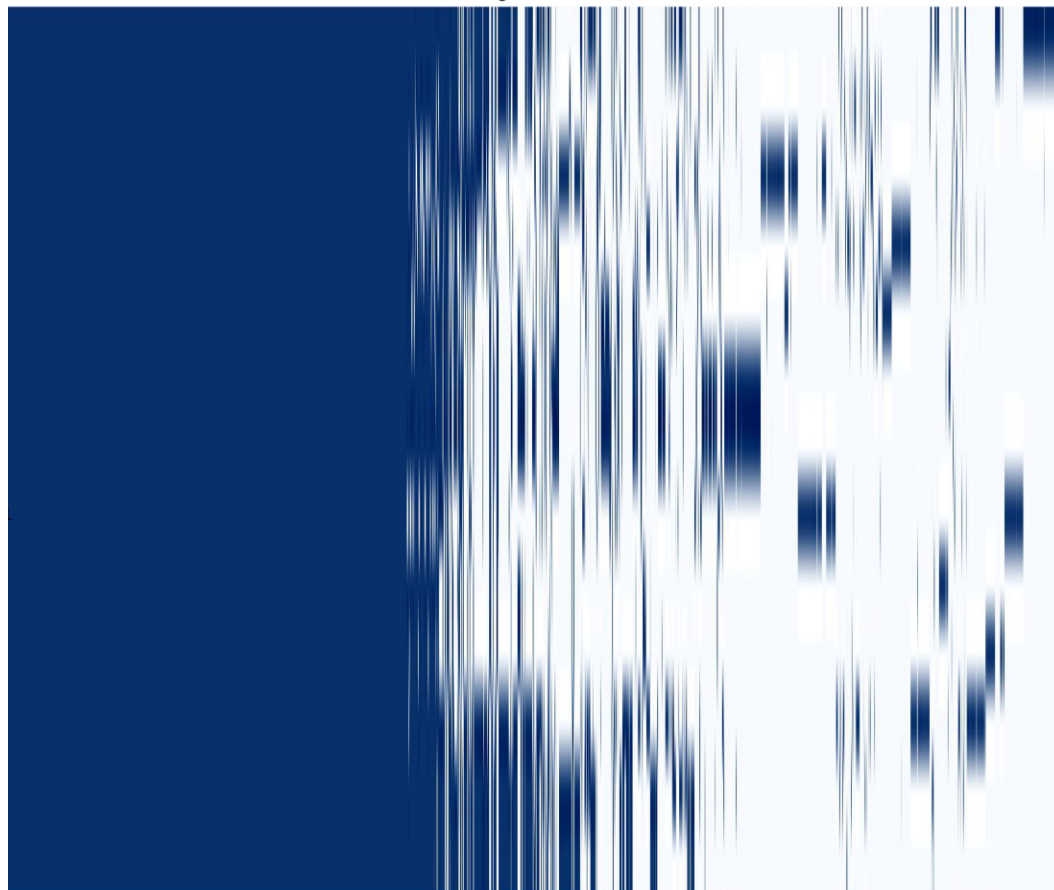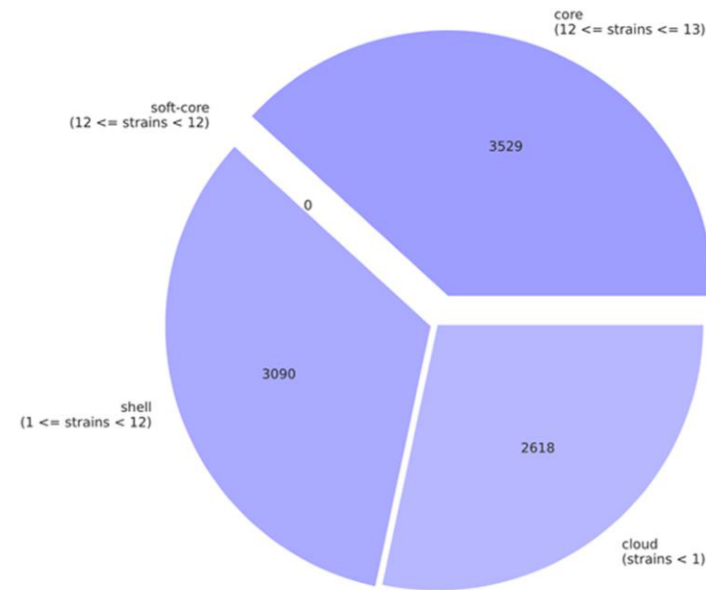

Supplement: Supplementary file 3 — Supplementary Figure 3. [file 41598_2024_55884_MOESM3_ESM.pdf]
