# Supplementary material for: Enterobacter asburiae ST229: an emerging carbapenemases producer
Source: Sci Rep. 2024 Mar 14;14:6220. doi: 10.1038/s41598-024-55884-y (PMC10940580; doi:10.1038/s41598-024-55884-y)
Supplement: Supplementary file 4 — Supplementary Table S1. [file 41598_2024_55884_MOESM4_ESM.docx]

**Supplementary Table S1:** number of SNPs detected

| **ID** | **6370** | **7108** | **ASM95260v1** | **ASM96582v1** | **ASM96600v1** | **PDT000533673.1** | **ASM2302326v1** | **ASM2315505v1** | **ASM2355900v1** | **ASM2375332v1** | **PDT001416897.1** | **ASM3058080v1** | **PDT001703140.1** |
| --- | --- | --- | --- | --- | --- | --- | --- | --- | --- | --- | --- | --- | --- |
| **6370** | 0 | 24 | 24479 | 16762 | 16764 | 1060 | 16646 | 24539 | 16694 | 16815 | 16417 | 16591 | 18685 |
| **7108** | 24 | 0 | 24471 | 16750 | 16752 | 1048 | 16634 | 24531 | 16682 | 16803 | 16405 | 16579 | 18673 |
| **ASM95260v1** | 24479 | 24471 | 0 | 20123 | 20121 | 24586 | 20189 | 443 | 20223 | 20191 | 20161 | 20216 | 23160 |
| **ASM96582v1** | 16762 | 16750 | 20123 | 0 | 26 | 16405 | 497 | 20153 | 467 | 346 | 664 | 488 | 5314 |
| **ASM96600v1** | 16764 | 16752 | 20121 | 26 | 0 | 16397 | 515 | 20151 | 485 | 364 | 682 | 506 | 5332 |
| **PDT000533673.1** | 1060 | 1048 | 24586 | 16405 | 16397 | 0 | 16293 | 24646 | 16341 | 16458 | 16064 | 16240 | 19225 |
| **ASM2302326v1** | 16646 | 16634 | 20189 | 497 | 515 | 16293 | 0 | 20241 | 108 | 229 | 637 | 211 | 5479 |
| **ASM2315505v1** | 24539 | 24531 | 443 | 20153 | 20151 | 24646 | 20241 | 0 | 20275 | 20219 | 20191 | 20268 | 23192 |
| **ASM2355900v1** | 16694 | 16682 | 20223 | 467 | 485 | 16341 | 108 | 20275 | 0 | 121 | 607 | 181 | 5449 |
| **ASM2375332v1** | 16815 | 16803 | 20191 | 346 | 364 | 16458 | 229 | 20219 | 121 | 0 | 486 | 302 | 5328 |
| **PDT001416897.1** | 16417 | 16405 | 20161 | 664 | 682 | 16064 | 637 | 20191 | 607 | 486 | 0 | 620 | 4978 |
| **ASM3058080v1** | 16591 | 16579 | 20216 | 488 | 506 | 16240 | 211 | 20268 | 181 | 302 | 620 | 0 | 5436 |
| **PDT001703140.1** | 18685 | 18673 | 23160 | 5314 | 5332 | 19225 | 5479 | 23192 | 5449 | 5328 | 4978 | 5436 | 0 |
