# Supplementary material for: Enterobacter asburiae ST229: an emerging carbapenemases producer
Source: Sci Rep. 2024 Mar 14;14:6220. doi: 10.1038/s41598-024-55884-y (PMC10940580; doi:10.1038/s41598-024-55884-y)
Supplement: Supplementary file 5 — Supplementary Legends. [file 41598_2024_55884_MOESM5_ESM.pdf]

Supplementary materials:

Supplementary Figure 1: PHYLOVIZ representation of all linked *E. asburiae* MLSTs available in PubMLST website.

Supplementary Figure 2: iTOL v6 graphical representation of SNPs-based phylogeny of 481 genomes obtained with parsnp. Blue = ST709; red = ST229; light blue = ST27.

Supplementary Figure 3. A: the gene presence/absence matrix of the 13 *E. asburiae* genomes with. **B:** pie-chart of the core and soft-core content.
